# Autophagy modulation by OmpA-targeted lead compounds restores autophagic flux, reduces biofilm, and enhances immune response in *Acinetobacter baumannii*-infected pulmonary cells

**DOI:** 10.1128/spectrum.00036-26

**Published:** 2026-06-09

**Authors:** Saroj Sharma, Vishvanath Tiwari

**Affiliations:** 1 Department of Biochemistry, Central University of Rajasthan 206414, Ajmer, India; Indian Institute of Technology (BHU) Varanasi, Varanasi, Uttar Pradesh, India

**Keywords:** *Acinetobacter baumannii*, outer membrane protein A, autophagy, autophagosome, confocal microscopy, real-time PCR, autophagic escape, drug design

## Abstract

*Acinetobacter baumannii* is an opportunistic pathogen known for causing severe nosocomial infections. Autophagy, a key host defense mechanism against invading pathogens, is modulated by the virulence factor OmpA. OmpA is also responsible for bacterial adherence to host cells. Targeting OmpA provides a potential strategy to counteract these autophagic disruptions. *In silico* analysis (molecular mechanics and molecular dynamics simulations) and experimental validation of identified small molecule lead against OmpA were performed. These leads were tested in A549 cells infected with *A. baumannii*, revealing significant autophagic modulations. mRNA expression analysis showed accumulation of the autophagy marker LC3B and suppression of elongation-associated genes upon infection. Confocal microscopy of mCherry-GFP-LC3-transfected cells demonstrated a reduced mCherry/GFP ratio, indicating downregulated autophagic flux during infection. Treatment with the leads reversed these effects, restoring autophagic activity to levels seen in uninfected cells. Additionally, SEM analysis confirmed that the leads inhibited OmpA-mediated biofilm formation. They also reduced the internalization of *A. baumannii* in A549 cells. The leads also enhanced IFN-γ and IL-13 expression while reducing IL-10, promoting immune clearance of *A. baumannii* via autophagy modulation and suppression of Treg functions. These findings suggest that the identified leads hold promise as autophagy modulators, potentially regressing *A. baumannii’s* ability to escape host defense.

**IMPORTANCE:**

Autophagic escape of *Acinetobacter baumannii* is mediated by OmpA; therefore, targeting OmpA represents a promising strategy to counteract autophagy disruption. The identified lead effectively reverses autophagy modulation, inhibits OmpA-mediated biofilm formation and bacterial internalization, and promotes a pro-clearance immune response.

## INTRODUCTION

*Acinetobacter baumannii*, a gram-negative opportunistic pathogen, causes pneumonia, meningitis, and various hospital-acquired infections (1). WHO has listed *A. baumannii* as a critical pathogen for which therapy is urgently required (2) due to its current resistance to most antibiotics and increased mortality and morbidity during hospital-acquired infections (3). During its interaction with the host, *A. baumannii* adheres to the host cells (4). One such factor is OmpA, which facilitates the adherence of *A. baumannii*, promotes its invasion into the host cells, and induces apoptosis (5, 6). OmpA disruption or knockout experiments have shown a substantial decrease in the establishment of virulence in host cells (7). High instances of severe pneumonia and bacteremia have been observed in infections caused by strains of *A. baumannii*, which express relatively higher amounts of OmpA (8). On the other hand, OmpA-mediated autophagy is induced by the MAPK/JNK signaling pathway in the hosts (9). Autophagy is the host’s defense mechanism against invading pathogens and is responsible for eliminating bacteria (9, 10). Host cells activate autophagy mechanisms to kill the pathogen, which involves LC3B as an important marker (11). OmpA is involved in the disruption of autophagosome maturation and their fusion with lysosomes in HeLa cells; hence, OmpA is associated with the induction of incomplete autophagy (9). It is shown that OmpA exerted its autophagy-suppressive effect through inhibition of CaMKK2 phosphorylation, and *ompA*-deletion mutant strain displayed considerably enhanced autophagy induction via the AMPK-ULK1 pathway (12).

The establishment of *A. baumannii* infections and persistence inside the host cells have been attributed to the manipulations of host autophagic machinery by *A. baumannii* (13). But until now, no specific mechanism for manipulating the autophagic pathway by *A. baumannii* has been established. Infectious pathogens manipulate autophagy at different stages of the autophagy process (14). Hence, we have monitored the autophagic escape of *A. baumannii* during infection via real-time PCR-based mRNA analysis, protein analysis via western blotting, and microscopic methods.

OmpA of *A. baumannii* has been reported for its role in autophagic escape (9); hence, this protein was targeted to inhibit autophagic escape of the pathogen. In this manuscript, the lead molecules were screened from the complete DrugBank database using an *in-silico* approach that showed good interaction with OmpA. The top three FDA-approved compounds were shortlisted. The ability of the screened lead to revert autophagic modulations during infection of *A. baumannii* has been investigated using different techniques. Along with it, OmpA has been reported to have diverse roles in the establishment of the virulence of *A. baumannii,* such as non-specific porin, monitoring the transport of molecules and facilitating the formation of the biofilm (15, 16). Hence, the efficacy of the screened lead has also been monitored for affecting other functions of OmpA to confirm their interaction. In addition to that, polyvinylpyrrolidone (PVP) has been already established for its efficient interaction with OmpA through an *in silico* approach (17). Therefore, the ability of PVP to revert the autophagic modulations mediated by *A. baumannii*, along with its efficiency in inhibiting other functions, has also been monitored.

## MATERIALS AND METHODS

### Culturing of the bacteria

*A. baumannii* (strain AB5075, Opaque Variant VIR-O) was cultured in Muller–Hinton Broth (Himedia) as per the previous laboratory protocol in an incubator shaker at 37°C (18). A single colony was used for the inoculation of the culture, and the overnight-grown culture was centrifuged and washed with PBS (1.8 mM KH_2_PO_4_, 2.7 mM KCl, 137 mM NaCl, and 10 mM Na_2_HPO_4_) before coinfection studies.

### Culture of A549 cell line

A549 cell lines were grown, and their culture was maintained as per the established laboratory protocol (18). A549 cells were passaged upon confluency and seeded in a 35 mm dish/six-well plates 24 h before infecting with *A. baumannii*.

### Infecting A549 cells with *A. baumannii*

PBS wash was given to the A549 cells, which were then trypsinized (trypsin-EDTA; 0.05%). The single-cell suspension was used for seeding at a density of 0.3 × 10^6^ in six-well plates (18). The overnight-grown culture of AB5075 was centrifuged for 5 min at 5,000*g*. The pellet was washed with PBS twice and finally dissolved in PBS. A549 cells were grown till a confluency of 90% and washed with PBS. Only DMEM (without FBS and antibiotics) was added to the cells, and the culture of AB5075 was added at 100:1 multiplicity of infection (MOI), and treatment with the screened leads and PVP was given at 16 µg/mL. The cells were further incubated with 5% CO_2_ at 37°C for 6 h post-infection.

### FITC labeling of *A. baumannii*

AB5075 culture was centrifuged for 10 min at 5,000 rpm. The pellet was resuspended in 0.1 M NaHCO_3_. To this suspension, 0.2 µL fluorescein isothiocyanate (FITC) was added (10 mg/mL) and incubated in the dark for 30 min. After incubation, it was centrifuged at 5,000 rpm for 10 min with three Hanks' Balanced Salt Solution washes. The pellet was finally dissolved in DMEM for infecting A549 cells.

### Adhesion and invasion assay

A549 cells were seeded 24 h before the adhesion and invasion assay. Then, A549 cells were infected at 100:1 MOI with *A. baumannii*. The infection was noticed under two conditions, first in the cell culture media supplemented with penicillin-streptomycin (1 mL in 100 mL cell culture media; P0781 Sigma), and second in the media that were used without any antibiotic. After the completion of the incubation of the treatment, the media of the A549 cells was discarded and thrice washed with PBS. Then, 100 μL 1× PBST was added to each well. After that, the cells were scraped, and this lysate was diluted in 1 mL PBS, which was further used to make serial dilutions as per the plating requirements, such as 1/100 and 1/1,000. A total of 5–10 μL of the dilutions was spread on Luria Agar (Muller Hinton Agar; Himedia) plates and incubated at 37°C overnight. The next day, the colonies were counted, and bar graphs were plotted for the same. For the invasion assay, gentamicin protection assays were performed (19, 20). The antibacterial effect of gentamicin was evaluated against *A. baumannii* at concentrations of 10, 25, 50, 100, 150, 200, 250, and 300 μg/mL. The results showed that gentamicin exhibited antibacterial activity, with the strongest effect observed at 300 μg/mL hence used for the Gentamicin protection assay (see Fig. S1 available at https://doi.org/10.6084/m9.figshare.32088876). After the completion of the infection, PBS wash was given to A549 cells, and 100 μL DMEM with 300 μg/mL gentamicin (Himedia) was added to the cells. After incubating for 4 h, two PBS washes were given to the cells. Again, 100 μL PBST was added to each well, and then the cells were scraped. This lysate was added to 1 mL PBS, and then the sample was spread on LA plates similar to the adhesion assay, and the colonies were counted for plotting the colony-forming unit (CFU) bar graphs.

### Gene expression analysis of autophagy-related genes

The total RNA of A549 cells was isolated using TRIzol Reagent (Invitrogen) from A549 cells using. Jenway Genova Nano nanodrop was used to estimate the concentration of RNA. For the synthesis of cDNA from 1 µg RNA, Verso cDNA synthesis Kit (ThermoScientific) was used. Quantitative real-time PCR with SYBR green as a fluorophore (Applied Biosystem real-time PCR mix) was performed in CFX96 Touch Real-Time PCR (Bio-Rad). Table S1 depicts the primers and their annealing temperatures (in °C) used in the present study (see Table S1 available at https://doi.org/10.6084/m9.figshare.32088876). Tables S2 and S3 depict the reaction mixture for real-time PCR and its reaction conditions (see Tables S2 and S3 available at https://doi.org/10.6084/m9.figshare.32088876). To analyze the changes in the expression of autophagy genes, 2^–∆∆Ct^ method was used.

### *In silico* selection of lead against *A. baumannii* outer membrane protein A (OmpA)

#### Homology modeling and its validation

For the selection of the homology templates, BLASTp was performed, and the sequence was confirmed using Query cover, percent identity, and *E*-value (21). Homology modeling is performed by uploading the sequence of OmpA (AXV53527.1) in the Phyre2 server (22). GalaxyWeb-based refining of the model was performed (23), and the best-refined model was selected. The model validation was done based on the Ramachandran Values and Verify3D (24), performed by submitting the refined model to the PSVS (Protein Structure Validation Suite) tool and ProSAweb (25, 26). The addition of hydrogen atoms and water molecules, along with assigning and minimization of charges and molecules, respectively, was performed until they reached RMSD (root mean square deviation) 0.3Ǻ using the protein preparation wizard module available on Schrödinger 10.3.

#### Virtual screening and molecular mechanics energies combined with generalized born and surface area continuum solvation calculations

The DrugBank database library, comprising around 500,000 drugs, was used to screen lead compounds. Before proceeding with virtual screening, preparation of the ligands was performed using the LIGPREP module, which involved adding hydrogen, ionization assignment for the ligands (pH 7.0 ± 2.0) using the option “ionizer” available at OPLS_2005 (Optimized Potential for Ligand) force field. The compounds were subjected to GLIDE-based high-throughput virtual screening docking (27). Standard Precision and Extra Precision (XP) docking were performed after this. The shortlisted compounds from XP docking were then used to predict the binding free energies, where a more negative value indicates higher stability of the complex. XP docking files were used for Prime-Molecular Mechanics Energies Combined with Generalized Born and Surface Area Continuum Solvation (MMGBSA) simulation. MMGBSA energy is more reliable than only the docking interactions, which ranks the analyzed compounds in a better way. The modeled structure of OmpA and the top three virtual screening hits were also uploaded to the HDOCK server (28).

#### Molecular dynamics simulation

GROMACS was used to perform the molecular dynamics simulation (MDS) for the top-ranked complex of protein-ligand retrieved by MMGBSA analysis (29), similar to our published protocol (30). PRODRG was used to prepare the topology of the drug (31). The solvation of the protein-ligand complex was set in the dodecahedron box using “editconf” feature of GROMACS. The charges of proteins were neutralized by the addition of oppositely charged ions (32, 33). Then, at a constant ensemble, the system was heated (constant volume, number of particles; NVT, and temperature) from 0 to 310 K over 100 ps. After that, it was subjected to an NPT ensemble (constant pressure, volume, and number of particles) at 1 atm pressure and 310 K over 100 ps.

### EtBr accumulation assay

To assess the impact of lead on membrane permeability and OmpA-related function, we monitored steady-state accumulation kinetics over a 60 min period (34). While initial rate assays (15) focus on immediate influx, this extended time-course was selected to capture the full equilibrium between passive diffusion and active efflux mechanisms. The mutant of OmpA has been reported to exhibit higher EtBr accumulation in bacteria compared to the wild type (15); therefore, an EtBr accumulation assay was performed to assess the function of OmpA. The overnight-grown culture of OD_600_ of 0.6 of *A. baumannii* was centrifuged at 10,000*g* for 5 min. The pellet was washed twice in PBS and finally diluted to an OD600 of 0.4. To this suspension, 0.4% of glucose was added to energize *A. baumannii* cells and keep efflux pumps active, so any increase in EtBr accumulation reflects OmpA or efflux pump inhibition rather than energy depletion or cell damage. They were further inoculated in 96-well plates and treated with the screened leads and PVP at different concentrations. A total of 0.5 µg/mL EtBr was added to the treated culture in each well. The fluorescence was observed at intervals of 2 min for 1 h (BioTek synergyH1 microplate reader). The excitation and emission wavelengths were 530 and 600 nm, respectively.

### Minimum inhibitory concentration determination using the microbroth dilution method

The minimum inhibitory concentration (MIC) of the screened leads was estimated using the microbroth dilution method. *Acinetobacter baumannii* AB5075 was grown in LB media at 37°C overnight with shaking. The OD600 of the culture was adjusted to 0.4. A total of 100 μL of this inoculum was added to a 96-well microtiter plate. The screened leads were added in a series of dilutions ranging from 1 to 256 μg/mL. Wells containing *A. baumannii* without the treatment of screened leads were taken as a control. The plate was incubated at 37°C for 6 h. The growth of the bacteria was compared by taking absorbance at 600 nm.

### Biofilm formation assay

A single colony of wild-type AB5075 was inoculated at 37°C overnight in tryptic soy broth supplemented with 1% glucose. The overnight culture was seeded into the wells of 96-well plates and incubated at 30°C for 48 h without shaking. For the quantification of the inhibiting effect of lead compound and PVP on biofilm formation, the seeded bacterial culture was treated with different doses. To quantify the biofilm formation after discarding the free-floating cells, 100 μL crystal violet (0.1% [wt/vol]) was used to stain the biofilm. Stained biofilms were dissolved in 33% glacial acetic acid and OD580 was taken using a BioTek synergyH1 microplate reader (30). Further confirmation was done by analyzing the biofilms under the scanning electron microscope (SEM). For SEM analysis, the biofilms were grown upon coverslips in 35 mm dishes. After 48 h, the biofilms were treated with different doses of the screened inhibitors and incubated for 24 h. After incubation, the biofilms were fixed with 2.5% glutaraldehyde and analyzed under SEM (ThermoScientific Apreo2s).

### Confocal microscopy analysis

For confocal microscopy analysis, A549 cells (coverslips inserted in 35 mm dishes and 80% confluent) were transfected with a dual-label plasmid (mcherryGFPLC3; Addgene). Lipofectamine 3000 Transfection Reagent (Invitrogen) based transfection was performed. According to the user manual, mcherryGFPLC3 (2,500 ng plasmid DNA with 3.75 µL of Lipofectamine 3000 and 5 µL of P3000 Reagent) was transfected in A549 cells. For visualize autophagic modulations under a confocal microscope, A549 cells (fixed with formaldehyde; 4%) were stained with DAPI (1 µg/mL; Himedia) and FM4-64 (5 µg/mL; Invitrogen). The fixed cells were mounted on slides using DPX mountant (SRL) and then analyzed under a confocal microscope (Leica). The excitation and emission wavelengths of DAPI are 350 nm and 410 nm, respectively; for GFP, it is 488 nm and 510 nm, respectively; for mcherry, it is 587 nm and 610 nm, respectively, and for FM4-64, it is 515 nm and 640 nm, respectively.

### Statistical analysis

Statistical analysis of the data was performed using MS Excel and GraphPad Prism.

## RESULTS

### *A. baumannii* infection modulates autophagy in A549 cells

**Fig 1:**
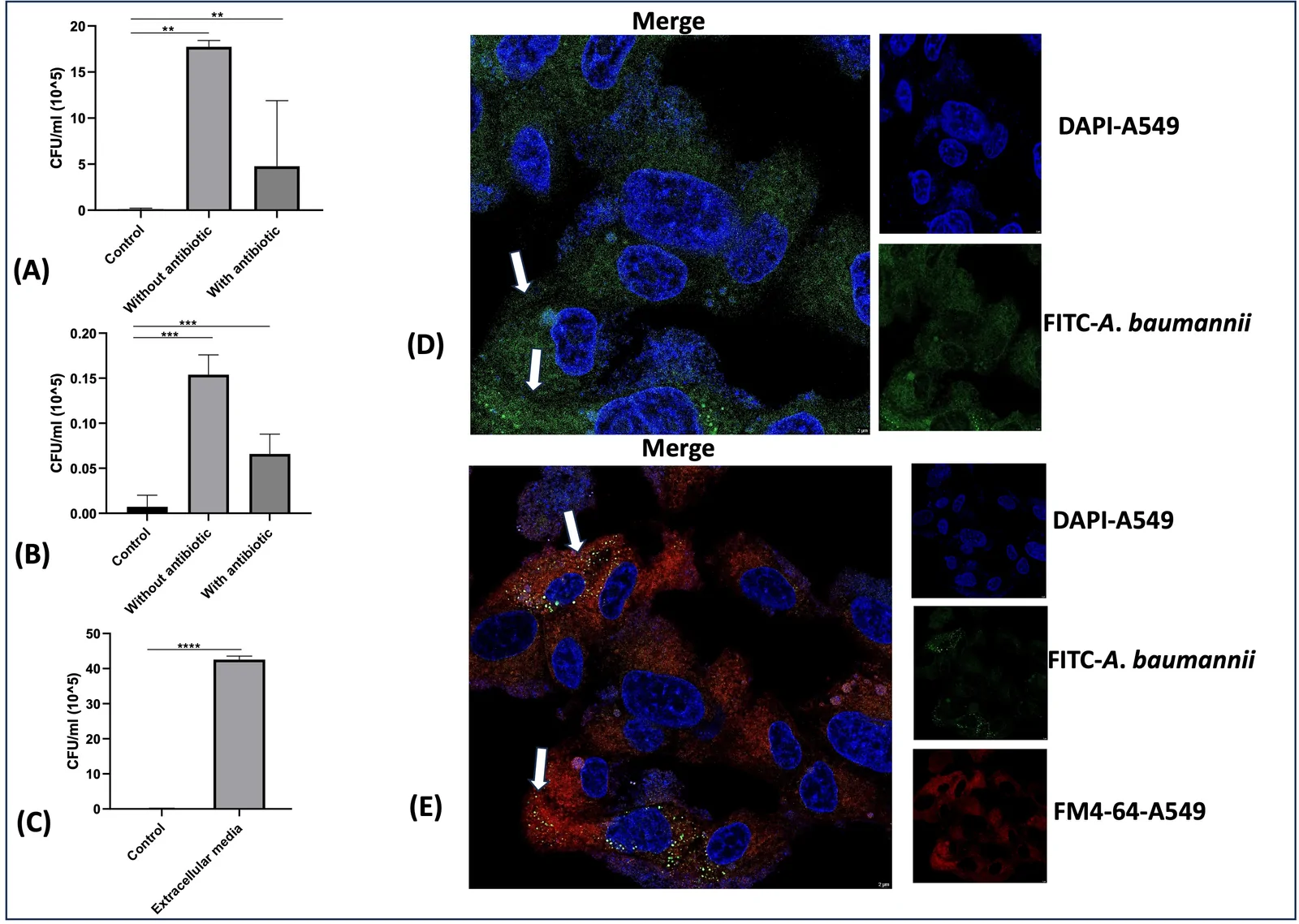
Adhesion and invasion of *A. baumannii* in A549 cells. Colony-forming unit analysis for the quantification of the (**A**) adhered (**B**) internalized *A. baumannii* with or without the presence of the antibiotic (penicillin-streptomycin; P0781 Sigma) of the human A549 cell culture media. The “control” refers only to A549 cells without any bacterial infection. (**C**) Colony-forming unit analysis of the growth of *A. baumannii* in the A549 cell culture media as compared to control without media. All the experiments were performed in triplicate, and for statistical analysis, ordinary one-way analysis of variance was performed, where error bars represent mean with SD; *P ≤ 0.05, **P ≤ 0.01, ***P ≤ 0.001, and ****P ≤ 0.0001 (**D**). Confocal microscopy analysis showing the internalization of *A. baumannii* inside A549 cells. DAPI represents the stained nucleus of the A549 cells. *A. baumannii* was labeled with FITC for internalization studies using confocal microscopy. (**E**) To further confirm the internalization of *A. baumannii*, FITC-labeled bacteria were used to infect A549 cells, and the membrane and nucleus of A549 cells were stained with FM4-64 and DAPI, respectively. Infection of A549 cells with *A. baumannii* was given at 100:1 MOI for 6 h. To distinguish between the adhered and invaded bacteria, the infected cells were treated with Gentamicin (300 µg/mL). The scale bar of the images is 2 μm. Images represent the best of the biological replicates.

**Fig 2:**
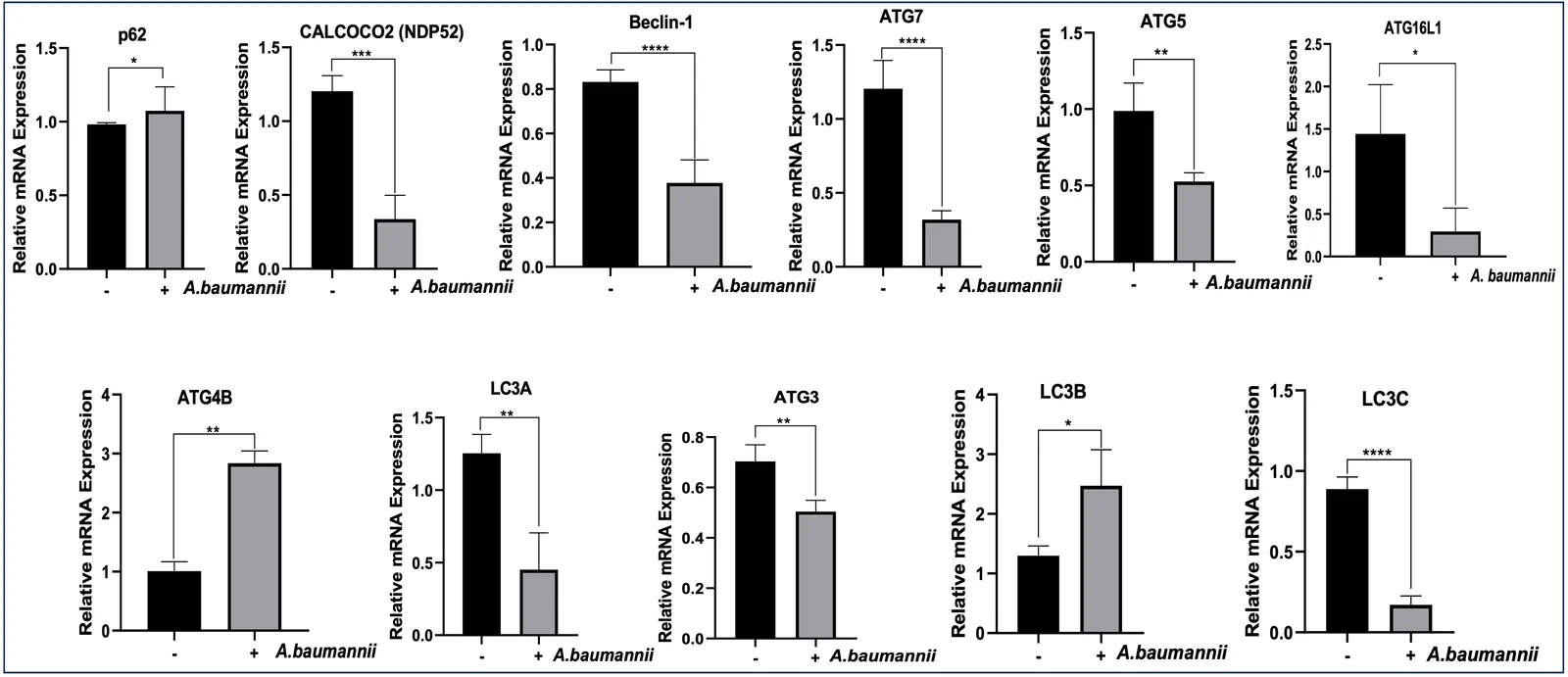
Real-time PCR data representing the relative mRNA expression analysis of autophagy-related genes. A549 cells were infected with *A. baumannii* at 100:1 MOI for 6 h for the control (uninfected) and infected with *A. baumannii*. All the experiments were performed in triplicate (minimum *n* = 3) and normalized with respect to 18S rRNA of each condition. Statistical analysis was performed with a *t*-test, where error bars represent the mean with SD. *P ≤ 0.05, **P ≤ 0.01, ***P ≤ 0.001, and ****P ≤ 0.0001.

*Acinetobacter baumannii* is known to cause lung infections; hence, pulmonary lung epithelial cell line A549 cells were infected with *A. baumannii* for this study. *A. baumannii* showed effective adhesion (Fig. 1A) and invasion (Fig. 1B) as confirmed by the CFU analysis in both the presence and absence of antibiotics in human cell culture media compared to the uninfected control. However, the growth of *A. baumannii* was more in the absence of the antibiotic of the cell culture media; hence, this condition was taken forward for further experiments. Similarly, significant growth was observed in the extracellular medium used for the A549 cells, in the absence of antibiotics after 6 h of infection (Fig. 1C). Furthermore, the internalization of *A. baumannii* in A549 cells was investigated using confocal microscopy analysis, which suggested internalization. The presence of FITC-labeled *A. baumannii* was observed around the nucleus (Fig. 1D). Similarly, the FM4-64-stained A549 cells again suggest the internalization of the FITC-labeled *A. baumannii*, as shown in Fig. 1E. These results suggest that *A. baumannii* efficiently internalizes inside the host cells. Further real-time PCR analysis of genes related to autophagy was performed to understand the autophagic modulations in A549 cells upon infection with *A. baumannii*. LC3B, a biomarker of autophagy, and the cargo receptor p62 were both upregulated at 6 h of *A. baumannii* infection (Fig. 2). Along with it, another cargo receptor, CALCOCO2, was downregulated after *A. baumannii* infection, indicating p62 and LC3B-mediated targeting of *A. baumannii* for autophagic clearance. Similarly, it is reported that in the OmpA mutant strain, levels of LC3 and p62 decreased compared to the WT strain, which indicated autophagic degradation; however, exogenous AbOmpA increased the accumulation of LC3-II and p62 by inhibiting autophagy-lysosomal fusion (12). In this study, the p62 and LC3B are upregulated, showing autophagic escape by *A. baumannii*. The bacterial clearance by autophagy involves the formation of phagophores, the creation of bacteria-containing vacuoles, the development of autophagosomes, the fusion of lysosomes, and the formation of autophagolysosomes. Hence, we have analyzed genes related to the different steps of autophagy by performing real-time PCR. It was observed that Beclin-1 (associated with the isolation of the phagophore membrane) and phagophore elongation (e.g., Atg5, Atg7, and Atg16l1) were downregulated after infection (Fig. 2). To investigate further the expression of different genes related to the LC3 processing, we have also monitored the expression of Atg4, which activates proLC3 into LC3A and localizes to the autophagic membrane in an LC3B-dependent manner (35). Atg4 also regulates the efficiency of autophagosome formation and influences the membrane recycling of LC3B. Atg7 (an E1-like enzyme) activates LC3A, which is later transferred to the E2-like enzyme Atg3 (36). At the site of lipidation, Atg3-LC3A conjugate is recruited via binding to the Atg16L1 complex. Here, the C-terminal of LC3A binds to the head of phosphatidylethanolamine via an amino bond, forming LC3B (11). It was observed that the infection of *A. baumannii* decreases the expression of Atg7, Atg5, Atg16L1, and Atg3, which might slow down the conversion of LC3A to LC3B. In contrast, Atg4B was significantly upregulated, indicating a higher recycling of LC3B (9). Furthermore, we have also observed a decrease in the level of LC3A, which may be attributed to the feedback mechanism of LC3B accumulation. These results showed that LC3B and p62 accumulated in the cells due to the increased load of clearance of infection of *A. baumannii* (9). This is further validated by a confocal microscopy study using the mCherry-GFP-LC3 reporter, which further confirms the result and is discussed in the coming section.

### Selection of virulence factor OmpA, modeling, and validation

**Fig 3:**
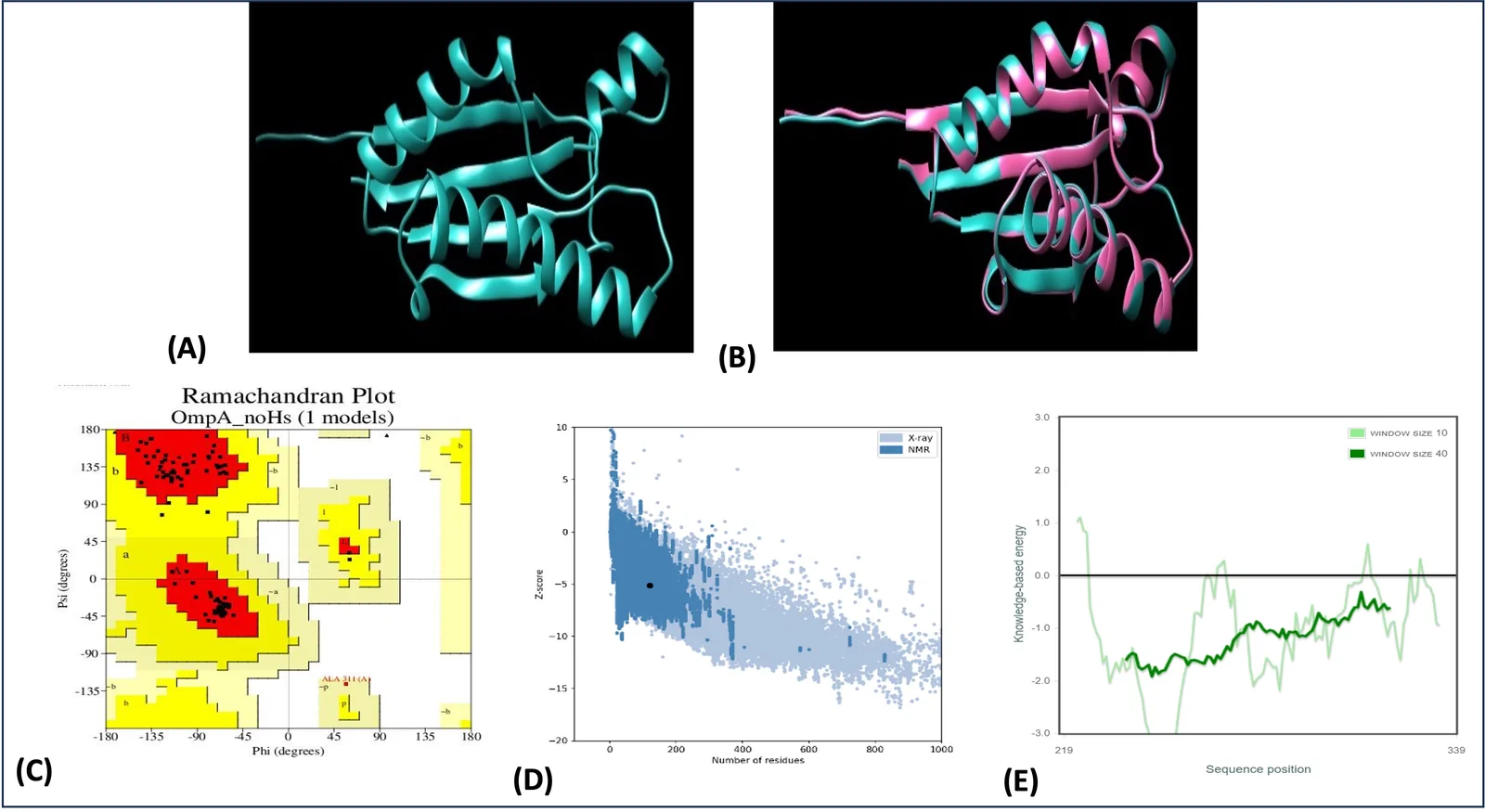
(**A**) Model OmpA (showing C-terminal domain only) constructed using Phyre2 server in which structure prediction is performed based on the selected sequence. Panel **B** represents the overlapped model of OmpA (showing C-terminal domain) obtained from Phyre2 (cyan colored) and the refined OmpA from GalaxyWeb (pink colored) (**C**) Ramachandran plot for modeled protein OmpA. (**D**) *Z*-score of −5.13 predicted by Prosa. (**E**) Peaks generated for modeled OmpA by Prosa.

Outer membrane protein, OmpA of *A. baumannii*, is known to be involved in incomplete induction of host autophagy (9) and modulates autophagy-related genes. Hence, we have selected OmpA protein of this bacterium to target its autophagic escape. OmpA is a highly conserved protein among *Acinetobacter* species (showing up to 100% identity and similarity in the Blastp results). OmpA was modeled using the intensive mode available on the Phyre2 web server Fig. 3A. Further refining of the models was performed using GalaxyWeb refine tool, and the model with the highest Ramachandran favored, lowest RMSD, MolProbity, and GDT-HA score was selected, i.e., model 1 (see Table S4 available at https://doi.org/10.6084/m9.figshare.32088876). Fig. 3B represents the overlapped structure of Phyre2 modeled OmpA (cyan color) with the GalaxyWeb refined model (pink color). PSVS validation was performed for the refined model, which shows that 98% of residues belong to the allowed region of the Ramachandran plot (Fig. 3C). The *Z*-score was −5.13 as observed via ProSAweb (here, lesser positive peaks are favored; Fig. 3D), and stable energy peaks of the model are shown in Fig. 3E.

### Selection of lead against OmpA using molecular docking and MMGBSA analysis

Molecular docking and MMGBSA analysis were performed to select leads against OmpA. Based on docking scores from both Glide and HDOCK analysis, the top virtual screening hits with lower MMGBSA energies were shortlisted (Table 1). The details of the binding free energies of the top virtual screening hits shortlisted by XP docking analysis have been provided in Table S5 available at https://doi.org/10.6084/m9.figshare.32088876. Among all the leads Mitoxantrone, Voglibose, and Ivermectin showed docking scores less than −9; hence, they were selected for further analysis. Our previous study showed that PVP shows good interaction with OmpA (17), but when this molecule was docked on the grid, it did not show a docking; hence, its monomer N-vinylpyrrolidone was docked in grid-based docking. This showed a favorable interaction, but a lesser docking score was observed. To further confirm the grid-based lead, we have performed global docking. The result of global docking showed that all four leads (Mitoxantrone [DB01204], Ivermectin [IVM; DB00602], Voglibose [DB04878], and PVP [DB11061]) showed good interaction with OmpA in global docking; hence, they were further selected for MDS.

**TABLE 1:** Top virtual screening hits from glide and global docking, along with their MMGBSA analysis^*b*^

Name of compound	MMGBSA dGbind (kcal/mol)	Glide score	Global docking
Mitoxantrone	−70.08	−10.28	−141.21
Ivermectin	−68.78	−9.34	−171.97
Voglibose	−51.82	−9.95	−82.38
PVP	−21.07	−2.689^*a*^	−250.78

^*a*^ Monomer of PVP was used for molecular docking in Glide.

^*b*^ Full details of all the selected leads are mentioned in Table S5 available at https://doi.org/10.6084/m9.figshare.32088876.

### Molecular dynamics simulation further confirmed the interaction of OmpA with leads

**Fig 4:**
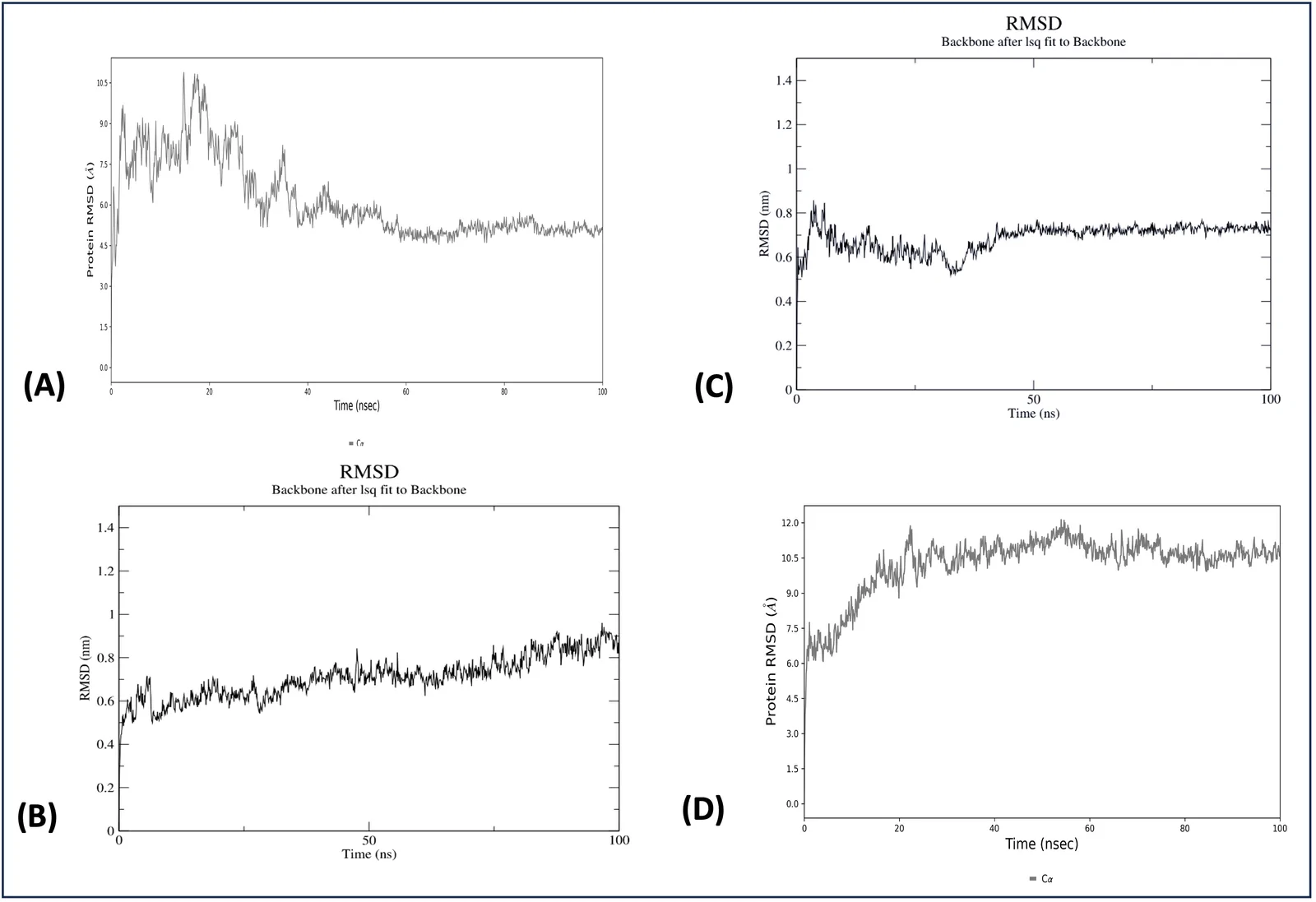
Molecular dynamics simulation showing RMSD of (**A**) OmpA-Mitoxantrone, (**B**) OmpA-IVM, (**C**) OmpA-Voglibose, and (**D**) OmpA-PVP depicting their RMSD.

The MDS analysis showed that the OmpA-Mitoxantrone complex is stable with RMSD of 6 Å at 100 ns. Similarly, OmpA-Ivermectin is stable with RMSD of 0.7 nm (7 Å), OmpA-Voglibose is stable with RMSD of 0.7 nm (7 Å), and OmpA-PVP is stable with RMSD of 10 Å. The RMSF of all proteins in the complex stage is significantly lower, further confirming the stability (see Fig. S2 available at https://doi.org/10.6084/m9.figshare.32088876). Hence, MDS analysis further confirmed the good interaction between the leads and the OmpA protein (Fig. 4). Subsequently, these were taken for experimental validation.

### Experimental validation of leads using the ethidium bromide accumulation assay

**Fig 5:**
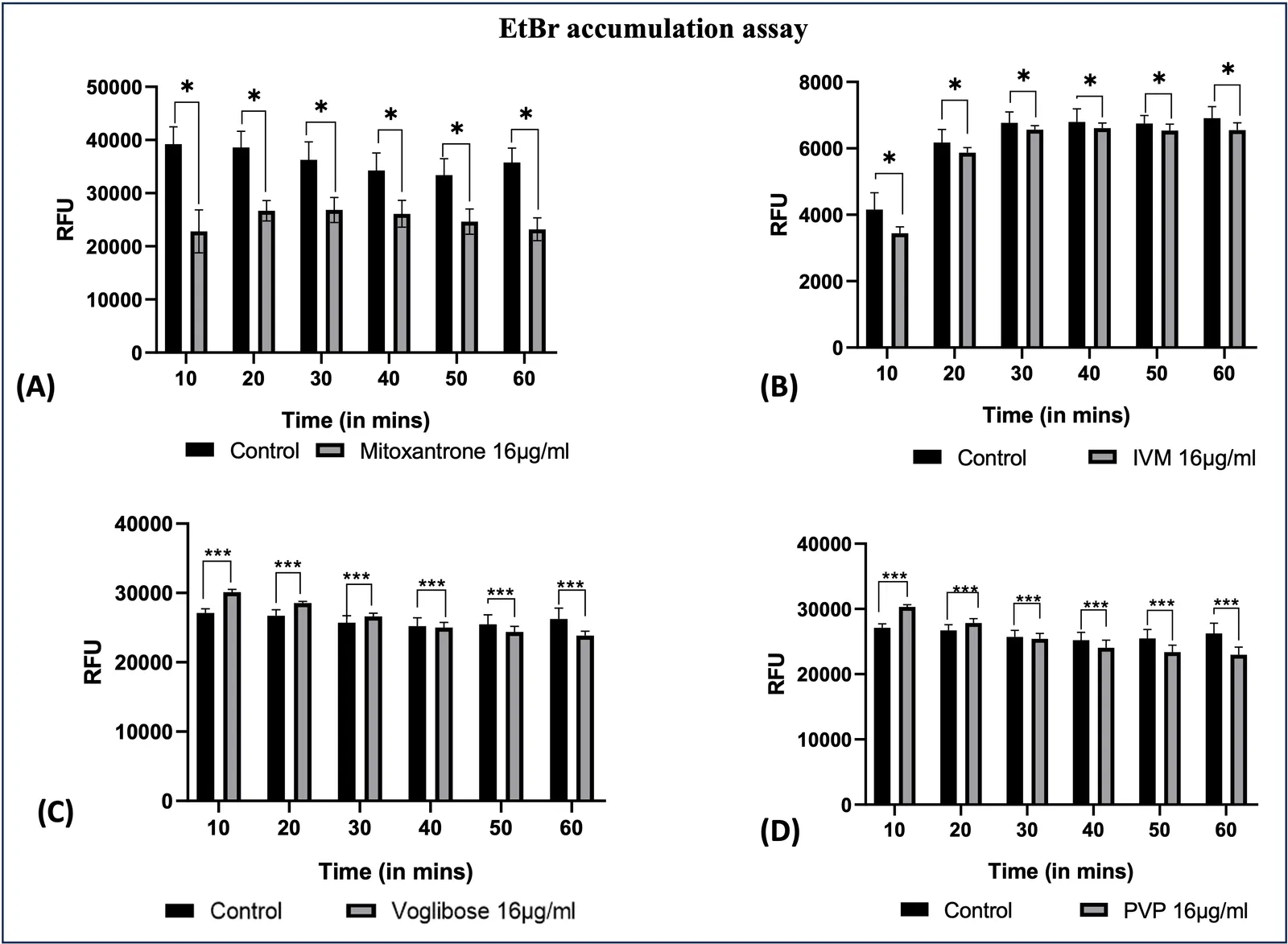
Graph representing (**A**) the decrease in the accumulation of EtBr in *A. baumannii* under treatment with mitoxantrone, (**B**) Ivermectin, (**C**) Voglibose, and (**D**) PVP (16 µg/mL) for 1 h. Each time point represents the mean of three independent fluorescent readings, and all the statistical analyses were performed using paired *t*-test, where error bars represent mean with SD; *P* < 0.05. The control group represents the bacterial culture without any treatment. To both groups, EtBr was added at *t* = 0, and fluorescence was observed at an interval of 2 min for an hour. *P ≤ 0.05 and ***P ≤ 0.001.

OmpA is a non-specific porin, known for virulence-associated properties to *A. baumannii* apart from beta-lactamases and various efflux pumps (37). To assess the activity of Mitoxantrone, IVM, Voglibose, and PVP against *A. baumannii*, we performed an ethidium bromide (EtBr) accumulation assay. *A. baumannii* was treated with different doses of the leads, and fluorescence was monitored (excitation and emission wavelengths are 530 and 600 nm, respectively) at a 2 min interval for a total time of 1 h. Our observations of the relative fluorescence unit against time (in minutes) showed a decrease in the accumulation of EtBr at 16 µg/mL of mitoxantrone (Fig. 5A). Similarly, Ivermectin also exhibited inhibition of EtBr accumulation at 16 µg/mL (Fig. 5B). However, PVP showed only a little inhibition of the accumulation of EtBr, whereas very little inhibiting effect was observed in the case of Voglibose (Fig. 5D and C, respectively). The EtBr accumulation assay under treatment with the screened leads at various concentrations is depicted in Supplementary data (see Fig. S3 to S6 available at https://doi.org/10.6084/m9.figshare.32088876). Based on the EtBr accumulation assay result, Voglibose has been dropped from further analysis.

### Treatment with leads inhibits the internalization of *A. baumannii* in A549 cells

**Fig 6:**
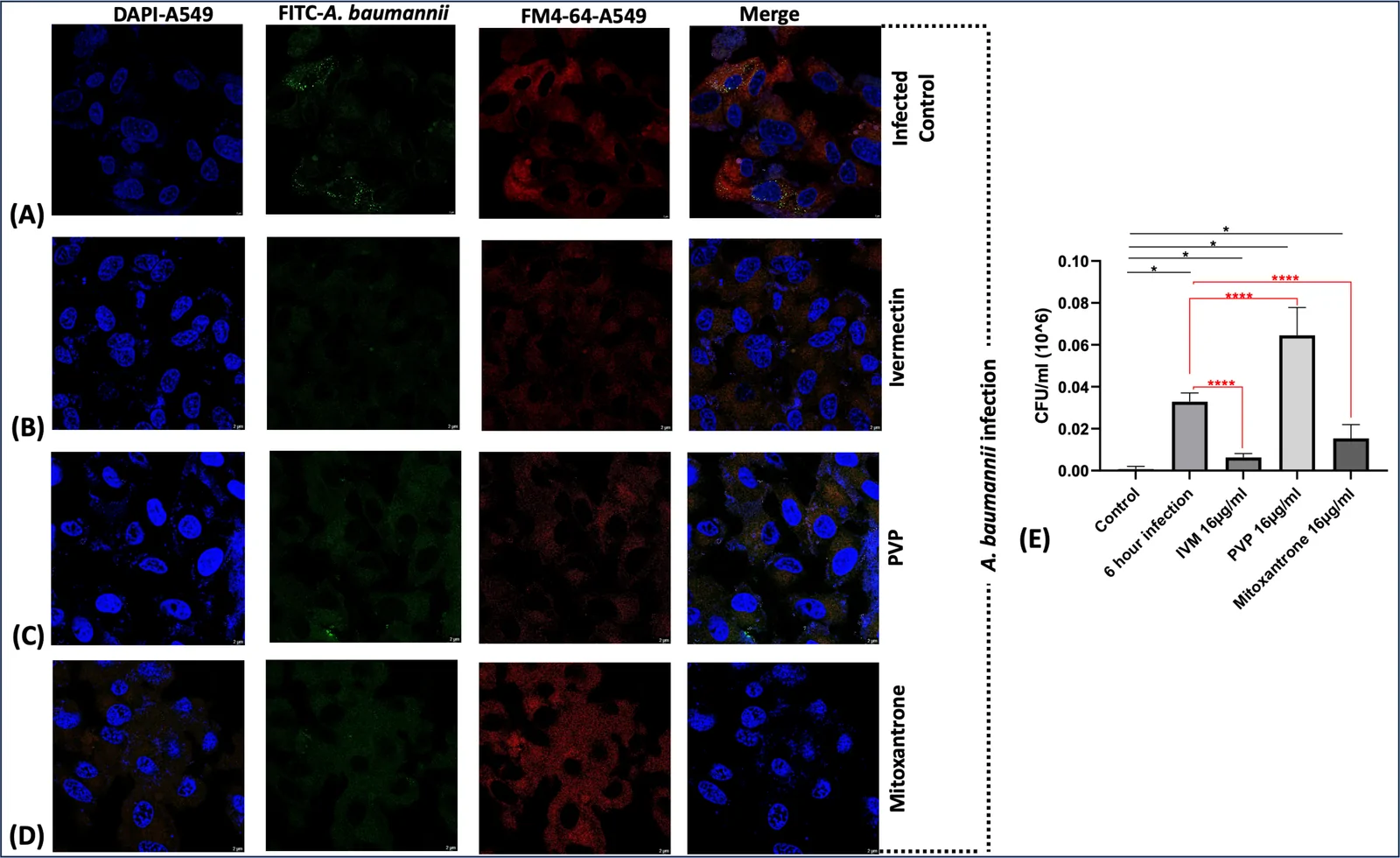
Confocal microscopy analysis showing the internalization of *A. baumannii* inside A549 cells treated with the screened leads (16 µg/mL). DAPI represents the stained nucleus of the A549 cells. *A. baumannii* was labeled with FITC for internalization studies using confocal microscopy. (**A**) Infected control, (**B–D**) Ivermectin, PVP, and Mitoxantrone treated infected A549 cells. To further confirm the internalization of *A. baumannii*, FITC-labeled bacteria were used to infect A549 cells, and the membrane and nucleus of A549 cells were stained with FM4-64 and DAPI, respectively. Infection of A549 cells with *A. baumannii* was given at 100:1 MOI for 6 h. To distinguish between the adhered and invaded bacteria, the infected cells were treated with Gentamicin (300 µg/mL) at 6 h and incubated for 4 h. The scale bar of the images is 2 μm. Images represent the best of the biological replicates. (**E**) CFU analysis was done to check the effect of screened leads on the internalization of *A. baumannii* in A549 cells infected at 100:1 MOI for 6 h. All the experiments were performed in triplicate, and for statistical analysis, one-way analysis of variance was performed, where error bars represent the mean with SD. *P ≤ 0.05, **P ≤ 0.01, ***P ≤ 0.001, and ****P ≤ 0.0001.

Upon treating A549 cells infected with *A. baumannii* with the screened leads at 16 µg/mL, there was a decrease in the green fluorescence associated with the FITC-labeled *A. baumannii*. This indicates that there was an inhibition in the internalization of *A. baumannii* under these treatments (Fig. 6A through D). The best inhibition of internalization was observed in the cases of Ivermectin and Mitoxantrone, compared to PVP. This was further evident by the CFU analysis (Fig. 6E), where both Ivermectin and mitoxantrone showed a decrease in the CFU, whereas PVP did not show any inhibitory effect on the internalization.

### Treatment with leads reverses the *A. baumannii*-mediated autophagic modulations

**Fig 7:**
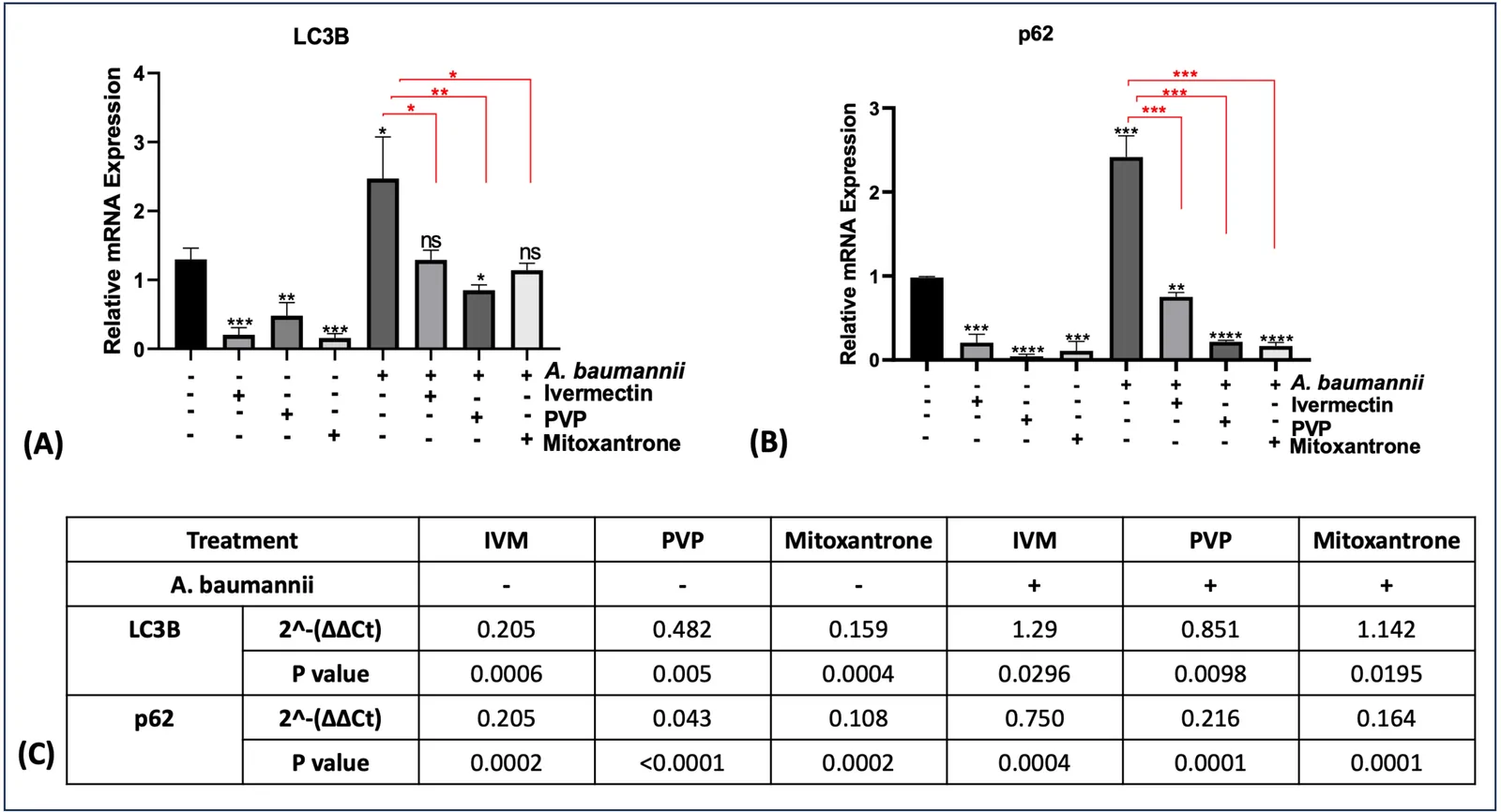
Real-time PCR data representing the relative expression analysis of autophagy-marker genes (**A**) LC3B and (**B**) p62. A549 cells were infected with *A. baumannii* at 100:1 MOI for 6 h for control (uninfected), infected with *A. baumannii* alone, IVM, Mitoxantrone, and PVP-treated *A. baumannii-*infected A549 cells. All experiments were performed in triplicate (minimum *n* = 3) and normalized to 18S rRNA. Statistical analysis was performed with *t*-test with respect to both the infected and uninfected control. Error bars represent the mean with SD. ns, not significant; *P ≤ 0.05, **P ≤ 0.01, ***P ≤ 0.001, and ****P ≤ 0.0001. The effect of only IVM was also monitored on the relative gene expression for LC3B and p62. (**C**) Table representing the 2^−(∆∆Ct)^ values along with the *P* values obtained from analysis using *t*-test with respect to the uninfected control for the treatments without infection and with infection (indicated with *P* values on the graphs) and infected control for the treatments in the presence of infection (indicated with *P* values in red).

Next, we studied the modulations of autophagy in A549 cells infected with *A. baumannii* and treated with Mitoxantrone, IVM, and PVP. For this, we have conducted a relative mRNA expression analysis of both important autophagy markers, LC3B and p62. Treatments resulted in the significant downregulation of LC3B and p62 compared to the infected control A549 cells (Fig. 7). An enhancement in the recruitment of cargo receptors for the clearance of *A. baumannii* after treating with leads and an increase in the autophagic flux could be the possible explanation of the results. Here, IVM reverted the autophagic modulations to the levels of the uninfected control, whereas both PVP and mitoxantrone showed more enhanced downregulation, indicating the rising autophagic flux. This suggests that the more effective clearance of *A. baumannii* may be achieved through the completion of autophagy. When the uninfected A549 cell lines were treated with lead, these genes were also downregulated. Hence, these leads may also have some other target in A549 cells, which needs further analysis. Furthermore, the reverting effects of Ivermectin on the autophagic escape of *A. baumannii* were analyzed using mRNA expression analysis of all the autophagy-related genes (see Fig. S7 available at https://doi.org/10.6084/m9.figshare.32088876).

### Confocal microscopy analysis of the autophagy modulations under different treatment conditions

**Fig 8:**
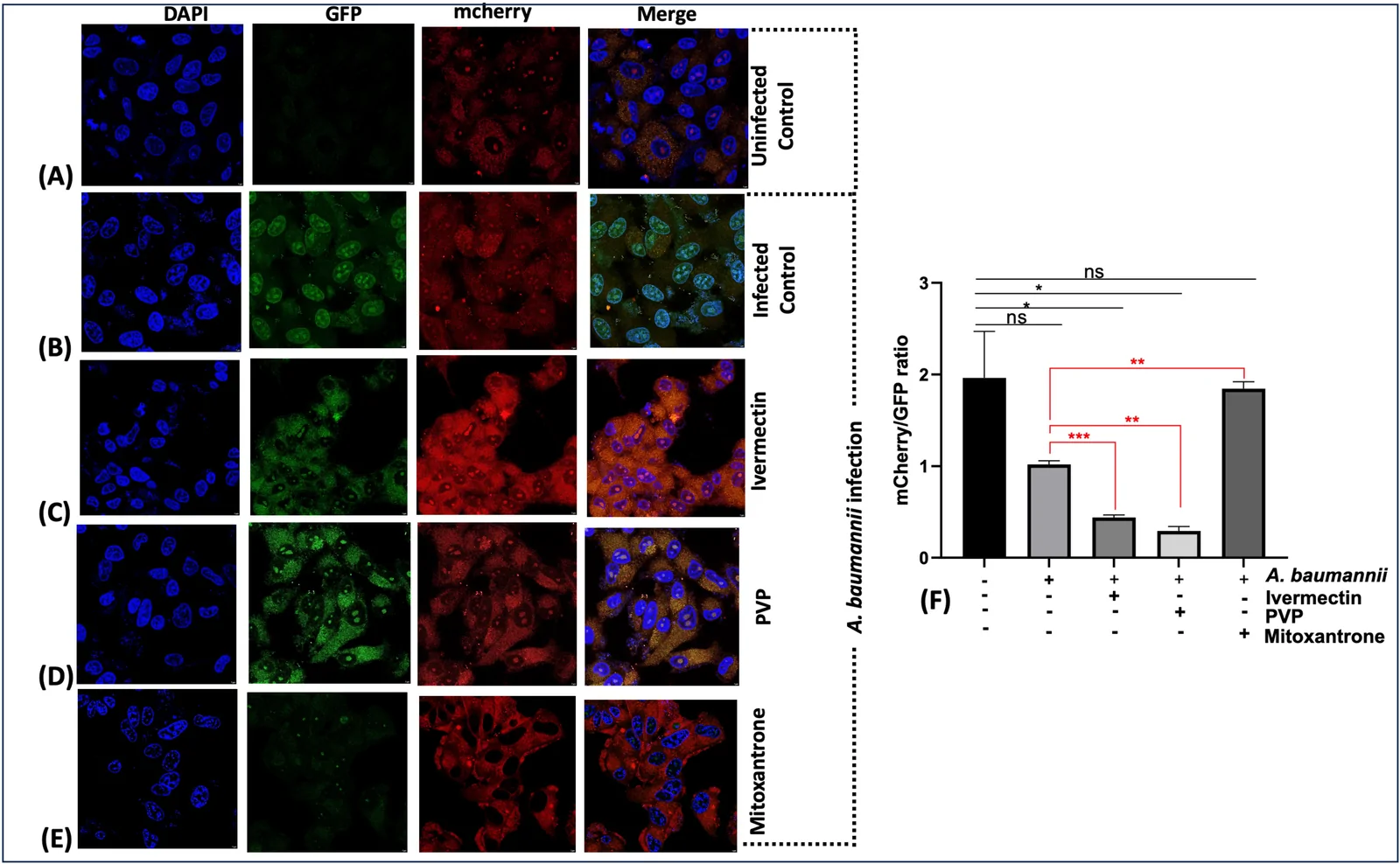
Confocal microscopy analysis of green (GFP) and red fluorescence (mcherry) in A549 cells transfected with mCherryGFP-LC3 under different treatments, which were infected with *A. baumannii* (100:1 MOI) for (**A**) Control, (**B**) 6 h infection with *A. baumannii* alone, and (**C**) treatment with IVM. (**D**) PVP and (**E**) Mitoxantrone (16 µg/mL) in *A. baumannii-*infected A549 cells for 6 h. (**F**) mcherry/GFP ratio representing the changes in the autophagic flux in various treatments, quantified using ImageJ software for analyzing the changes in fluorescence during different treatment conditions. Images represent the best out of the biological replicates at a scale bar of 2 μm. All statistical analyses were performed using multiple *t*-test; *P* < 0.05 and error bars indicate mean with SD. ns, not significant; *P ≤ 0.05.

Using confocal microscopy, the effect of the treatments of the screened leads on A549 cells infected with *A. baumannii* has been monitored to observe the modulations of autophagy (Fig. 8). But before that, we have analyzed the changes in the autophagic flux in total bacterial load (both extracellular and intracellular) as compared to only intracellular bacteria. The results showed that total bacterial load showed a decrease in the mcherry/GFP ratio as compared to the only intracellular bacteria (see Fig. S8 available at https://doi.org/10.6084/m9.figshare.32088876), as evident by the increased yellow fluorescence. The results indicate that *A. baumannii* infection modulates autophagy of A549 cells, as both GFP and mCherry fluorescence started rising compared to the uninfected control (Fig. 8A and B). However, *A. baumannii* infection decreases the turnover of mcherry from GFP, which might indicate the inhibition of autophagic completion as compared to the uninfected control (Fig. 8F). Furthermore, we have analyzed the effects of the selected leads in reverting these modulations in *A. baumannii*-infected cells. When the infected cells were treated with IVM, GFP was upregulated as compared to the mcherry indicating the enhanced cargo recruitment and promotion of autophagy (Fig. 8C). Whereas, in the case of PVP treatment, mcherry was highly downregulated, indicating that PVP treatment is promoting the completion of autophagy, and higher fusion of autophagosomes and lysosomes is taking place (Fig. 8D). Under the treatment of mitoxantrone the GFP fluorescence remained almost similar to the infected control, whereas the rise in the mcherry fluorescence indicated that mitoxantrone promotes the maturation of autophagosomes and hence increases the flux by increasing the fusion of the autophagosomes with lysosomes (Fig. 8E). The ratio of mcherry/GFP was calculated for comparing the GFP and mcherry fluorescence in different treatment conditions to understand the changes in the autophagic flux as represented in Fig. 8F. These results altogether indicate that out of the selected leads, mitoxantrone has higher efficiency in promoting the completion of autophagy. As mitoxantrone reverts the ratio (mcherry/GFP) to the level of uninfected control. The lower the ratio, the lower the autophagic flux, and vice versa.

### Lead inhibits the initiation and maturation of biofilms in AB5075

**Fig 9:**
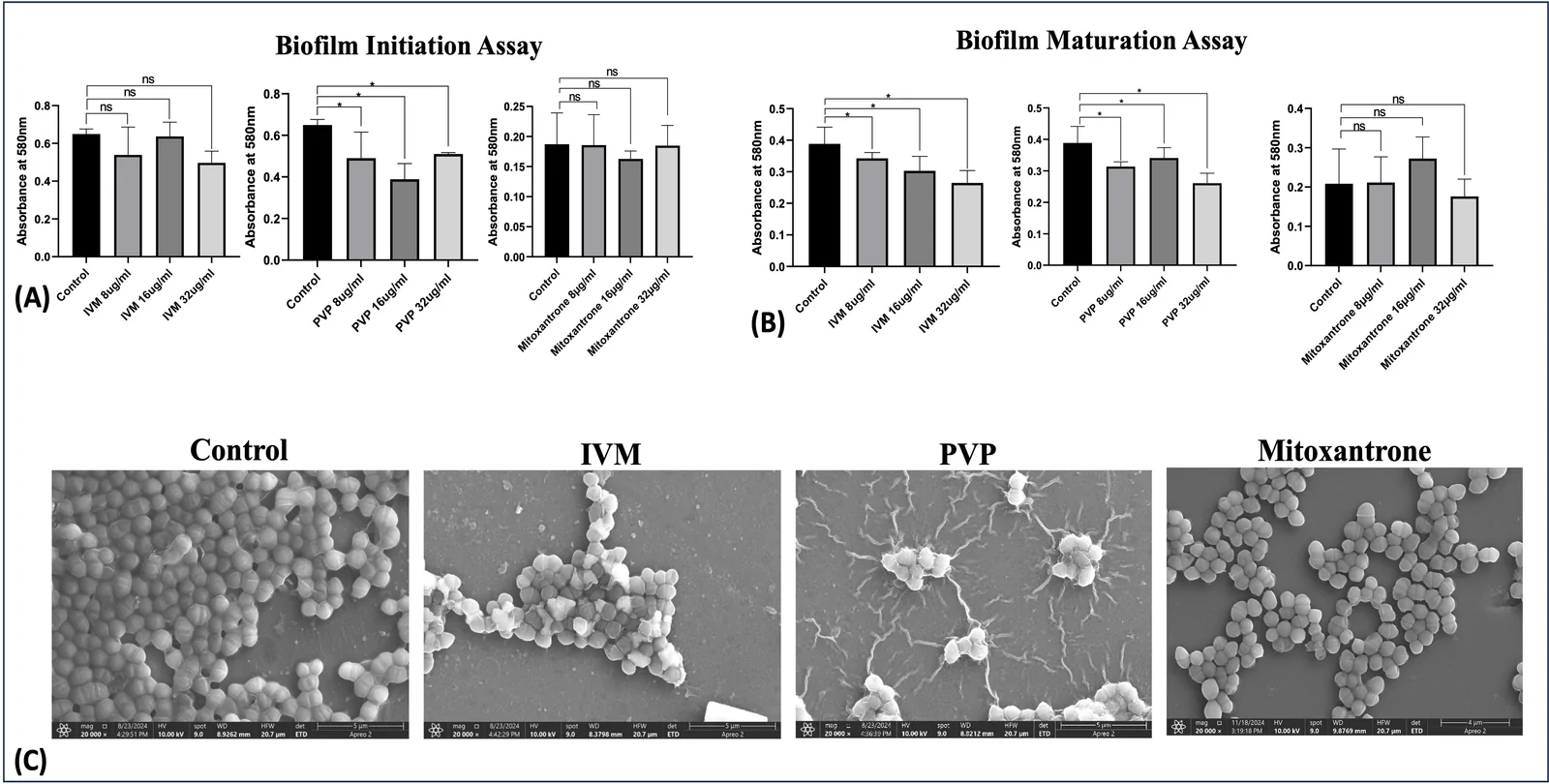
Bar graph representing the inhibitory effect of IVM, PVP, and mitoxantrone on (**A**) biofilm initiation and (**B**) biofilm maturation ability of *A. baumannii* at different concentrations. The experiment was performed in triplicate, and statistical analysis was performed using one-way analysis of variance; *P* < 0.05, where error bars indicate mean with SD. (**C**) The effect of screened leads upon biofilm formation was further confirmed using SEM. The scale bar of the Control, IVM, and PVP was 5 μm, and for mitoxantrone, it was 4 μm at 20,000× magnification for each image. ns, not significant; *P ≤ 0.05.

OmpA is known to play its role in the formation of biofilms, hence favoring the colonization of *A. baumannii* at both biotic and abiotic surfaces. Here, we also monitored the effects of the screened leads to inhibit other functions of OmpA. However, before that, it was important to check the inhibitory effects of the screened leads against *A. baumannii*, as we only aimed to assess the efficacy of the screened leads against the biofilm-forming ability of *A. baumannii*. The MIC results confirmed that the screened leads were non-antibacterial against *A. baumannii* (see Fig. S9 available at https://doi.org/10.6084/m9.figshare.32088876). Thereafter, we assessed the activity of leads in targeting the initial attachment and colonization (initiation of biofilm) and transformation of the biofilms into more complex structures and expenditure (maturation of biofilm). Treatment with IVM and PVP (8–32 µg/mL) showed a significant decrease in biofilm formation (Fig. 9A and B), whereas a lesser inhibitory role of mitoxantrone was observed. This suggests that IVM and PVP have an OmpA-inhibiting role as OmpA promotes the formation of biofilm. Our results were further confirmed using SEM analysis (Fig. 9C), where both the treatment groups (IVM and PVP) showed lesser biofilm formation compared to the untreated control, and mitoxantrone showed little changes in the inhibition of biofilms.

### Leads modulate the inflammatory markers during infection of *A. baumannii*

**Fig 10:**
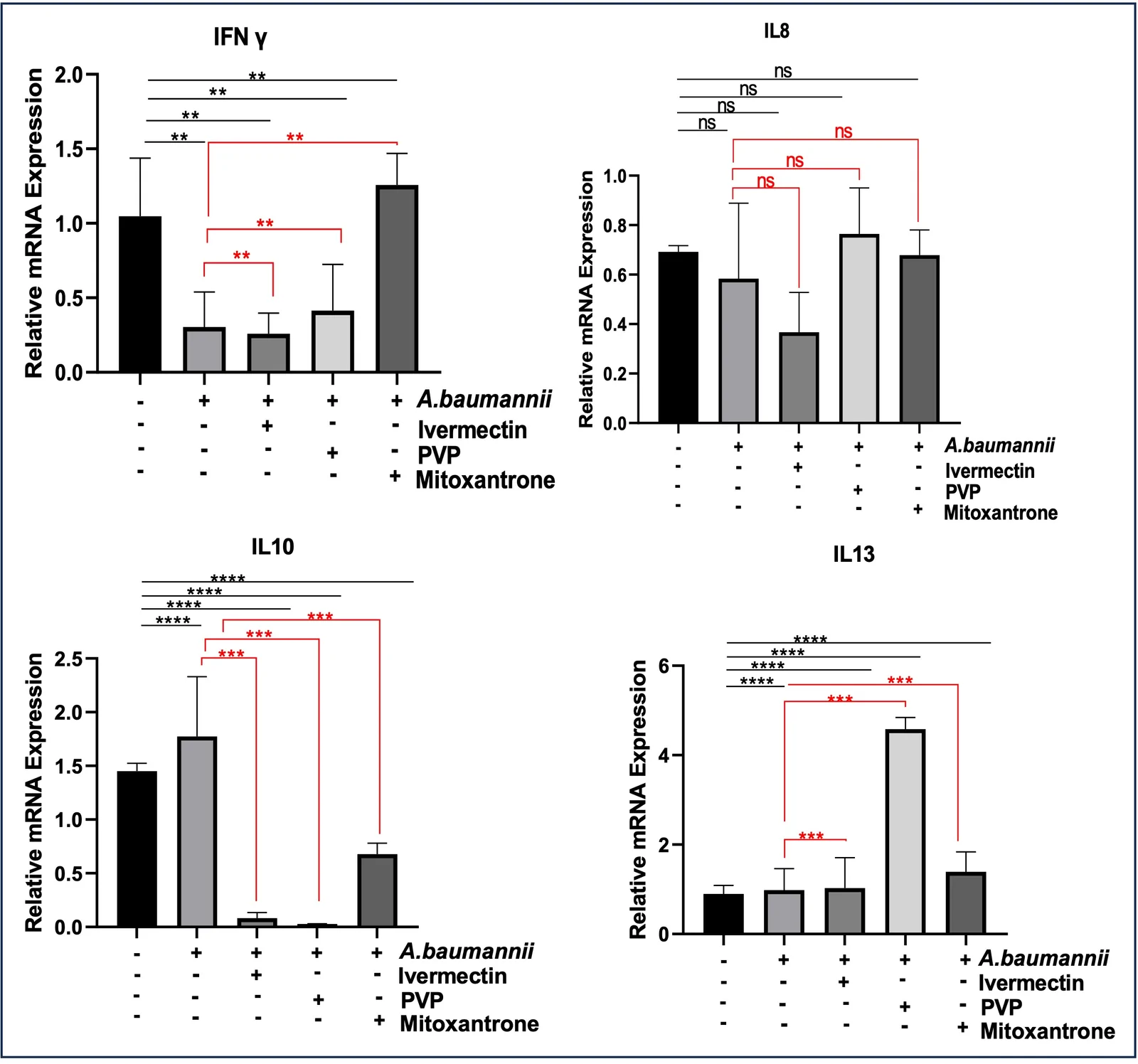
Real-time PCR data representing the relative expression analysis of inflammatory response genes (IFN-γ, IL-8, IL-10, and IL-13). A549 cells were infected with *A. baumannii* at 100:1 MOI for 6 h. Uninfected control A549 cells were compared with infected *A. baumannii* alone, and IVM, Mitoxantrone, and PVP-treated *A. baumannii*-infected A549 cells. All the experiments were performed in triplicate (minimum *n* = 3) and normalized to 18S rRNA in each condition. Statistical analysis was performed with one-way analysis of variance, where error bars represent the mean with SD. ns, not significant; **P ≤ 0.01, ***P ≤ 0.001, and ****P ≤ 0.0001.

The infection with *A. baumannii* can also be correlated with the pro-inflammatory markers like IFN-γ and IL-8 and anti-inflammatory markers like IL-10 and IL-13 (38). IFN-γ is involved in the TH1 response and activates macrophages to kill intracellular bacteria and enhance antigen presentation (39). IL-8 recruits neutrophils for improved clearance of bacteria from the site of infection (40). IL-10 promotes Treg, hence enhancing bacterial persistence by limiting immune clearance (41). IL-13 promotes a TH2 response and modulates the macrophage response to bacterial antigens (42). The downregulation of IFN-γ after infection may be a strategy to suppress macrophage activation and clearance. A decrease in IL-8 reduces neutrophils, but the non-significant changes indicate that neutrophils are not involved as the bacteria are inside the endosomes. Upregulation of IL-10 after infection prevents excessive inflammation and bacterial persistence. Similarly, IL-13 upregulation promotes a TH2 response against pathogens (Fig. 10). Recently, it has been observed that *A. baumannii* uses IL-13 for the dissemination or spread of infection in the lungs of mice (43).

As a previous microbiological study suggests, treatment prevents autophagic escape and alters the immune response. Comparing the expression analysis of IFN-γ showed that mitoxantrone and PVP enhance its expression as compared to the infected cells, suggesting more bacterial clearance. A similar result was expected in IL8, but no significant changes were observed. All the leads reduce IL-10, which indicates that enhanced clearance of the pathogen eliminates the need for Treg function to balance the immune response. Leads enhance IL-13 (TH2 response) and promote the clearance of pathogens, but their direct role requires further investigation. All the gene expression profiles together suggest clearance of *A. baumannii,* hence supporting their role in promoting autophagy.

## DISCUSSION

The prevalence of the multidrug-resistant *A. baumannii* has become a matter of serious concern. There have been various instances in the literature showing that *A. baumannii* induces incomplete autophagy via its OmpA protein in the host cells (9, 12). The evolutionarily conserved mechanism of autophagy maintains cellular homeostasis and has been attributed to the clearance of pathogenic bacteria. In this study, OmpA of *A. baumannii* was targeted through *in silico* approaches and selected lead molecules (Mitoxantrone, IVM, and Voglibose; all had a docking score <–9) and another PVP (docking score –2.689 as its monomer was docked with OmpA) from our previous study (17). MMGBSA scores of –70.08, –68.78, and −51.82 indicate efficient binding of IVM, mitoxantrone, and Voglibose with OmpA, respectively. The MMGBSA score of PVP was −21.07. The MDS result shows a stable interaction between the selected leads and OmpA. IVM, a macrolide, has proven effective against the biofilms of *A. baumannii* and has been recommended as a combinatorial drug to treat the infections of *A. baumannii* (44). Lim and Omansen et al. have shown in separate studies the efficacy of IVM against *Mycobacterium tuberculosis* and *M. ulcerans*, as well as methicillin-resistant strains of *S. aureus* (45–47). Whereas Voglibose is an α-glucosidase inhibitor important during the treatment of type II diabetes (48). Mitoxantrone is an anti-cancer agent and is reported to have antibacterial activity as well as an immunomodulatory activity (49). EtBr accumulation assay with the DB04878 (Voglibose) inhibitor showed no promising decline in the inhibition of accumulation of EtBr in the bacteria, and hence was not taken forward for further studies at mRNA and protein levels. Ivermectin, PVP, and Mitoxantrone also showed a decline in the internalization of *A. baumannii* in A549 cells. Treatment with these leads showed a decrease in the accumulation of autophagy-associated genes LC3B and p62 in the *A. baumannii*-infected cells. This suggests the completion of autophagy for the clearance of invading bacteria. However, the uninfected cells also showed a downregulation of these genes, indicating that the screened leads might have some other targets in A549 cells. Among the leads, mitoxantrone was the best to revert the downregulated mcherry/GFP ratio to the levels of the uninfected control, as infection showed a decrease in the autophagic flux (decreased mcherry/GFP ratio). The effect of IVM, mitoxantrone, and PVP on biofilm formation by *A. baumannii* was also analyzed. A decline in the formation of biofilm in both IVM and PVP-treated groups was detected compared to the control, whereas mitoxantrone showed no effect. Furthermore, IFN-γ, IL-8, IL-10, and IL-13-mediated immune responses were also analyzed. It was observed that the treatment with mitoxantrone and PVP reverses the downregulation of IFN-γ to the uninfected levels, indicating enhanced clearance of the bacteria. IL-8 exhibited the same changes, but the reversion was non-significant. The leads suppress the expression of IL-10, indicating no Treg involvement and an enhanced clearance of the pathogen has occurred. All the results together suggest that these three leads, Mitoxantrone, IVM, and PVP, could be used to study further effects; however, Mitoxantrone did not show inhibition of biofilm, a key function of OmpA. Hence, IVM and PVP could be considered as better, but all three leads can be further investigated for possible inhibition of OmpA. The next step in this study would be the isolation and purification of OmpA from *A. baumannii*, followed by the validation of its interaction with the lead molecule. Future studies should focus on detailed biochemical and biophysical validation of the direct interactions between identified leads and OmpA, including binding affinity and structural analyses. This would provide us with a clearer picture of all the processes leading to the manipulation of host autophagy and the establishment of infections.

## Data Availability

All the data are available in the manuscript, as well as in Supplementary Figures S1 to S9 and Tables S1 to S5, which are available online via link (https://doi.org/10.6084/m9.figshare.32088876).

## References

[1] Maragakis LL, Perl TM. 2008. *Acinetobacter baumannii*: epidemiology, antimicrobial resistance, and treatment options. Clin Infect Dis 46:1254–1263. doi:10.1086/529198 18444865

[2] Boucher HW, Talbot GH, Bradley JS, Edwards JE, Gilbert D, Rice LB, Scheld M, Spellberg B, Bartlett J. 2009. Bad bugs, no drugs: no ESKAPE! an update from the Infectious Diseases Society of America. Clin Infect Dis 48:1–12. doi:10.1086/595011 19035777

[3] Chuang YC, Chang SC, Wang WK. 2010. High and increasing Oxa-51 DNA load predict mortality in *Acinetobacter baumannii* bacteremia: implication for pathogenesis and evaluation of therapy. PLoS One 5:e14133. doi:10.1371/journal.pone.0014133 21152436 PMC2994729

[4] Lee JC, Koerten H, van den Broek P, Beekhuizen H, Wolterbeek R, van den Barselaar M, van der Reijden T, van der Meer J, van de Gevel J, Dijkshoorn L. 2006. Adherence of *Acinetobacter baumannii* strains to human bronchial epithelial cells. Res Microbiol 157:360–366. doi:10.1016/j.resmic.2005.09.011 16326077

[5] Choi CH, Hyun SH, Lee JY, Lee JS, Lee YS, Kim SA, Chae J-P, Yoo SM, Lee JC. 2008. *Acinetobacter baumannii* outer membrane protein A targets the nucleus and induces cytotoxicity. Cell Microbiol 10:309–319. doi:10.1111/j.1462-5822.2007.01041.x 17760880

[6] Choi CH, Lee JS, Lee YC, Park TI, Lee JC. 2008. *Acinetobacter baumannii* invades epithelial cells and outer membrane protein A mediates interactions with epithelial cells. BMC Microbiol 8:216. doi:10.1186/1471-2180-8-216 19068136 PMC2615016

[7] Gaddy JA, Tomaras AP, Actis LA. 2009. The *Acinetobacter baumannii* 19606 OmpA protein plays a role in biofilm formation on abiotic surfaces and in the interaction of this pathogen with eukaryotic cells. Infect Immun 77:3150–3160. doi:10.1128/IAI.00096-09 19470746 PMC2715673

[8] Sánchez-Encinales V, Álvarez-Marín R, Pachón-Ibáñez ME, Fernández-Cuenca F, Pascual A, Garnacho-Montero J, Martínez-Martínez L, Vila J, Tomás MM, Cisneros JM, Bou G, Rodríguez-Baño J, Pachón J, Smani Y. 2017. Overproduction of outer membrane protein A by *Acinetobacter baumannii* as a risk factor for nosocomial pneumonia, bacteremia, and mortality rate increase. J Infect Dis 215:966–974. doi:10.1093/infdis/jix010 28453834

[9] An Z, Huang X, Zheng C, Ding W. 2019. Acinetobacter baumannii outer membrane protein A induces HeLa cell autophagy via MAPK/JNK signaling pathway. Int J Med Microbiol 309:97–107. doi:10.1016/j.ijmm.2018.12.004 30606692

[10] Cadwell K, Abraham C, Bel S, Chauhan S, Coers J, Colombo MI, Davis JR, Hofius D, Nguyen HTT, Ogawa M, Roy CR, Shao F, Shizukuishi S, Stallings CL, Szczesna M, Taylor G, Thurston TL, Watson R, Wileman T, Xu Y, Zamboni DS. 2025. Autophagy and bacterial infections. Autophagy Rep 4:2542904. doi:10.1080/27694127.2025.2542904 40910070 PMC12407897

[11] Noda T, Fujita N, Yoshimori T. 2009. The late stages of autophagy: how does the end begin? Cell Death Differ 16:984–990. doi:10.1038/cdd.2009.54 19424283

[12] Woo K, Kim DH, Park H-S, Oh MH, Lee JC, Choi CH. 2025. *Acinetobacter baumannii* OmpA hinders host autophagy via the CaMKK2-reliant AMPK-pathway. mBio 16:e0336924. doi:10.1128/mbio.03369-24 39998213 PMC11980379

[13] Parra-Millán R, Guerrero-Gómez D, Ayerbe-Algaba R, Pachón-Ibáñez ME, Miranda-Vizuete A, Pachón J, Smani Y. 2018. Intracellular trafficking and persistence of *Acinetobacter baumannii* requires transcription factor EB. mSphere 3:e00106-18. doi:10.1128/mSphere.00106-18 29600279 PMC5874439

[14] Sharma S, Tiwari M, Tiwari V. 2021. Therapeutic strategies against autophagic escape by pathogenic bacteria. Drug Discov Today 26:704–712. doi:10.1016/j.drudis.2020.12.002 33301978

[15] Sugawara E, Nikaido H. 2012. OmpA is the principal nonspecific slow porin of *Acinetobacter baumannii*. J Bacteriol 194:4089–4096. doi:10.1128/JB.00435-12 22636785 PMC3416538

[16] Nie D, Hu Y, Chen Z, Li M, Hou Z, Luo X, Mao X, Xue X. 2020. Outer membrane protein A (OmpA) as a potential therapeutic target for *Acinetobacter baumannii* infection. J Biomed Sci 27:26. doi:10.1186/s12929-020-0617-7 31954394 PMC6969976

[17] Sharma S, Tiwari V. 2024. Polyvinylpyrrolidone capped silver nanoparticles enhance the autophagic clearance of *Acinetobacter baumannii* from human pulmonary cells. Discov Nano 19:154. doi:10.1186/s11671-024-04107-4 39313578 PMC11420407

[18] Tiwari V, Tiwari M, Solanki V. 2017. Polyvinylpyrrolidone-capped silver nanoparticle inhibits infection of carbapenem-resistant strain of *Acinetobacter baumannii* in the human pulmonary epithelial cell. Front Immunol 8:973. doi:10.3389/fimmu.2017.00973 28861082 PMC5561010

[19] Kaneko M, Emoto Y, Emoto M. 2016. A simple, reproducible, inexpensive, yet old-fashioned method for determining phagocytic and bactericidal activities of macrophages. Yonsei Med J 57:283. doi:10.3349/ymj.2016.57.2.283 26847277 PMC4740517

[20] Sycz G, Di Venanzio G, Distel JS, Sartorio MG, Le N-H, Scott NE, Beatty WL, Feldman MF. 2021. Modern *Acinetobacter baumannii* clinical isolates replicate inside spacious vacuoles and egress from macrophages. PLoS Pathog 17:e1009802. doi:10.1371/journal.ppat.1009802 34370792 PMC8376066

[21] Verma P, Tiwari V. 2018. Targeting outer membrane protein component AdeC for the discovery of efflux pump inhibitor against AdeABC efflux pump of multidrug resistant *Acinetobacter baumannii*. Cell Biochem Biophys 76:391–400. doi:10.1007/s12013-018-0846-5 29926429

[22] Kelley LA, Mezulis S, Yates CM, Wass MN, Sternberg MJE. 2015. The Phyre2 web portal for protein modeling, prediction and analysis. Nat Protoc 10:845–858. doi:10.1038/nprot.2015.053 25950237 PMC5298202

[23] Ko J, Park H, Heo L, Seok C. 2012. GalaxyWEB server for protein structure prediction and refinement. Nucleic Acids Res 40:W294–7. doi:10.1093/nar/gks493 22649060 PMC3394311

[24] Eisenberg D, Lüthy R, Bowie JU. 1997. VERIFY3D: assessment of protein models with three-dimensional profiles. Methods Enzymol 277:396–404. doi:10.1016/s0076-6879(97)77022-8 9379925

[25] Bhattacharya A, Tejero R, Montelione GT. 2007. Evaluating protein structures determined by structural genomics consortia. Proteins 66:778–795. doi:10.1002/prot.21165 17186527

[26] Wiederstein M, Sippl MJ. 2007. ProSA-web: interactive web service for the recognition of errors in three-dimensional structures of proteins. Nucleic Acids Res 35:W407–10. doi:10.1093/nar/gkm290 17517781 PMC1933241

[27] Friesner RA, Murphy RB, Repasky MP, Frye LL, Greenwood JR, Halgren TA, Sanschagrin PC, Mainz DT. 2006. Extra precision glide: docking and scoring incorporating a model of hydrophobic enclosure for protein−ligand complexes. J Med Chem 49:6177–6196. doi:10.1021/jm051256o 17034125

[28] Yan Y, Tao H, He J, Huang S-Y. 2020. The HDOCK server for integrated protein-protein docking. Nat Protoc 15:1829–1852. doi:10.1038/s41596-020-0312-x 32269383

[29] Abraham MJ, Murtola T, Schulz R, Páll S, Smith JC, Hess B, Lindahl E. 2015. GROMACS: high performance molecular simulations through multi-level parallelism from laptops to supercomputers. SoftwareX 1–2:19–25. doi:10.1016/j.softx.2015.06.001

[30] Tiwari V, Patel V, Tiwari M. 2018. In-silico screening and experimental validation reveal L-adrenaline as anti-biofilm molecule against biofilm-associated protein (Bap) producing *Acinetobacter baumannii*. Int J Biol Macromol 107:1242–1252. doi:10.1016/j.ijbiomac.2017.09.105 28964839

[31] Schüttelkopf AW, van Aalten DMF. 2004. PRODRG: a tool for high-throughput crystallography of protein-ligand complexes. Acta Crystallogr D Biol Crystallogr 60:1355–1363. doi:10.1107/S0907444904011679 15272157

[32] Rajendran V. 2016. Structural analysis of oncogenic mutation of isocitrate dehydrogenase 1. Mol Biosyst 12:2276–2287. doi:10.1039/c6mb00182c 27194485

[33] Rajendran V, Gopalakrishnan C, Sethumadhavan R. 2018. Pathological role of a point mutation (T315I) in BCR-ABL1 protein-A computational insight. J Cell Biochem 119:918–925. doi:10.1002/jcb.26257 28681927

[34] Richmond GE, Chua KL, Piddock LJV. 2013. Efflux in *Acinetobacter baumannii* can be determined by measuring accumulation of H33342 (bis-benzamide). J Antimicrob Chemother 68:1594–1600. doi:10.1093/jac/dkt052 23467176 PMC3682688

[35] Zhou Y, Wang Z, Huang Y, Bai C, Zhang X, Fang M, Ju Z, Liu B. 2022. Membrane dynamics of ATG4B and LC3 in autophagosome formation. J Mol Cell Biol 13:853–863. doi:10.1093/jmcb/mjab059 34562084 PMC8800521

[36] Koukourakis MI, Kalamida D, Giatromanolaki A, Zois CE, Sivridis E, Pouliliou S, Mitrakas A, Gatter KC, Harris AL. 2015. Autophagosome proteins LC3A, LC3B and LC3C have distinct subcellular distribution kinetics and expression in cancer cell lines. PLoS One 10:e0137675. doi:10.1371/journal.pone.0137675 26378792 PMC4574774

[37] Smani Y, Fàbrega A, Roca I, Sánchez-Encinales V, Vila J, Pachón J. 2014. Role of OmpA in the multidrug resistance phenotype of *Acinetobacter baumannii*. Antimicrob Agents Chemother 58:1806–1808. doi:10.1128/AAC.02101-13 24379205 PMC3957889

[38] García-Patiño MG, García-Contreras R, Licona-Limón P. 2017. The immune response against *Acinetobacter baumannii*, an emerging pathogen in nosocomial infections. Front Immunol 8:441. doi:10.3389/fimmu.2017.00441 28446911 PMC5388700

[39] Chen W. 2020. Host innate immune responses to *Acinetobacter baumannii* infection. Front Cell Infect Microbiol 10:486. doi:10.3389/fcimb.2020.00486 33042864 PMC7521131

[40] Sarshar M, Behzadi P, Scribano D, Palamara AT, Ambrosi C. 2021. *Acinetobacter baumannii*: an ancient commensal with weapons of a pathogen. Pathogens 10:387. doi:10.3390/pathogens10040387 33804894 PMC8063835

[41] Kang M-J, Jang A-R, Park J-Y, Ahn J-H, Lee T-S, Kim D-Y, Lee M-S, Hwang S, Jeong Y-J, Park J-H. 2020. IL-10 protects mice from the lung infection of *Acinetobacter baumannii* and contributes to bacterial clearance by regulating STAT3-mediated MARCO expression in macrophages. Front Immunol 11:270. doi:10.3389/fimmu.2020.00270 32153580 PMC7047127

[42] Yan Z, Yang J, Hu R, Hu X, Chen K. 2016. *Acinetobacter baumannii* infection and IL-17 mediated immunity. Mediators Inflamm 2016:9834020. doi:10.1155/2016/9834020 26977122 PMC4762998

[43] Palmer LD, Traina KA, Juttukonda LJ, Lonergan ZR, Bansah DA, Ren X, Geary JH, Pinelli C, Boyd KL, Yang TS, Skaar EP. 2024. Dietary zinc deficiency promotes *Acinetobacter baumannii* lung infection via IL-13 in mice. Nat Microbiol 9:3196–3209. doi:10.1038/s41564-024-01849-w 39548344 PMC11800279

[44] Yamabe K, Arakawa Y, Shoji M, Onda M, Miyamoto K, Tsuchiya T, Akeda Y, Terada K, Tomono K. 2020. Direct anti-biofilm effects of macrolides on *Acinetobacter baumannii*: comprehensive and comparative demonstration by a simple assay using microtiter plate combined with peg-lid. Biomed Res 41:259–268. doi:10.2220/biomedres.41.259 33268670

[45] Ashraf S, Chaudhry U, Raza A, Ghosh D, Zhao X. 2018. *In vitro* activity of ivermectin against *Staphylococcus aureus* clinical isolates. Antimicrob Resist Infect Control 7:27. doi:10.1186/s13756-018-0314-4 29468054 PMC5819080

[46] Lim LE, Vilchèze C, Ng C, Jacobs WR Jr, Ramón-García S, Thompson CJ. 2013. Anthelmintic avermectins kill Mycobacterium tuberculosis, including multidrug-resistant clinical strains. Antimicrob Agents Chemother 57:1040–1046. doi:10.1128/AAC.01696-12 23165468 PMC3553693

[47] Omansen TF, Porter JL, Johnson PDR, van der Werf TS, Stienstra Y, Stinear TP. 2015. In-vitro activity of avermectins against *Mycobacterium ulcerans*. PLoS Negl Trop Dis 9:e0003549. doi:10.1371/journal.pntd.0003549 25742173 PMC4351077

[48] Oh TJ, Yu JM, Min KW, Son HS, Lee MK, Yoon KH, Song YD, Park JY, Jeong IK, Cha BS, Kim YS, Baik SH, Kim IJ, Kim DM, Kim SR, Lee KW, Park JH, Lee IK, Park TS, Choi SH, Park SW. 2019. Efficacy and safety of voglibose plus metformin in patients with type 2 diabetes mellitus: a randomized controlled trial. Diabetes Metab J 43:276–286. doi:10.4093/dmj.2018.0051 30604594 PMC6581551

[49] da Silva RAG, Wong JJ, Antypas H, Choo PY, Goh K, Jolly S, Liang C, Tay Kwan Sing L, Veleba M, Hu G, Chen J, Kline KA. 2023. Mitoxantrone targets both host and bacteria to overcome vancomycin resistance in *Enterococcus faecalis*. Sci Adv 9:eadd9280. doi:10.1126/sciadv.add9280 36812322 PMC9946351

